# Sudden Unexpected Death and the Mammalian Dive Response: Catastrophic Failure of a Complex Tightly Coupled System

**DOI:** 10.3389/fphys.2019.00097

**Published:** 2019-02-19

**Authors:** Frank F. Vincenzi

**Affiliations:** ^1^Department of Pharmacology, University of Washington, Seattle, WA, United States; ^2^Pharmacological Information and Consultation Service, Arlington, WA, United States

**Keywords:** diving bradycardia, fatal drowning, long QT syndrome, normal accidents, sudden cardiac death, sudden infant death syndrome, sudden unexpected death in epilepsy

## Abstract

In tightly coupled complex systems, when two or more factors or events interact in unanticipated ways, catastrophic failures of high-risk technical systems happen rarely, but quickly. Safety features are commonly built into complex systems to avoid disasters but are often part of the problem. The human body may be considered as a complex tightly coupled system at risk of rare catastrophic failure (sudden unexpected death, SUD) when certain factors or events interact. The mammalian dive response (MDR) is a built-in safety feature of the body that normally conserves oxygen during acute hypoxia. Activation of the MDR is the final pathway to sudden cardiac (SCD) in some cases of sudden infant death syndrome (SIDS), sudden unexpected death in epilepsy (SUDEP), and sudden cardiac death in water (SCDIW, fatal drowning). There is no single cause in any of these death scenarios, but an array of, unanticipated, often unknown, factors or events that activate or interact with the mammalian dive reflex. In any particular case, the relevant risk factors or events might include a combination of genetic, developmental, metabolic, disease, environmental, or operational influences. Determination of a single cause in any of these death scenarios is unlikely. The common thread among these seemingly different death scenarios is activation of the mammalian dive response. The human body is a complex tightly coupled system at risk of rare catastrophic failure when that “safety feature” is activated.

## Complex Tightly Coupled Technical Systems

In a book titled, *Normal Accidents: Living with High-Risk Technologies*, Perrow, described what he called “normal accidents” (Perrow, 1999). To avoid multiple references to several quotes from that book, I refer in brackets [p.xx] to the page numbers on which they appear. According to Perrow, “normal accidents” are inherent catastrophic failures that are bound to occur (albeit, rarely) in highly complex technical systems (e.g., nuclear power plants, space travel, etc.). Catastrophic failure is viewed as the potential “…to take the lives of hundreds of people in one blow, or to shorten or cripple the lives of thousands or millions more” [p. 3].

In the 1984 edition of *Normal Accidents*, Perrow noted, that “…the probability of a nuclear plant meltdown is not one chance in a million a year, but more like one chance in the next decade” [p. 4]. The Chernobyl catastrophic accident happened on 25–26 April 1986. Other nuclear power plant failures, airline disasters, and technological misfortunes examined by Perrow have been covered in the media. In reconstructing the series of events that lead to such failures we rely critically on the details of the design (e.g., original blueprints), construction, and intended functions of each of the components of highly technological systems, as well as their proper operation.

What follows is not the result of a systematic review, but a bringing together of ideas from three seemingly disparate avenues viewed together through the lens provided by Perrow. Perrow refers to “the interactive complexity” of the system in which minor failures interact in an unanticipated way that quickly leads to catastrophic failure. He also defines “tightly coupled systems” as those in which, “…processes happen very fast and can’t be turned off, the failed parts cannot be isolated from other parts, or there is no other way to keep the production going safely” [p. 4]. In many cases, operators of such complex technological systems are not able to understand the unanticipated problem. This may lead to operator error and catastrophic failure. The essential point is that sooner or later in complex systems, unanticipated interactions will appear and catastrophic failures will occur–with or without operator error.

Efforts to prevent failures have prompted inclusion of various safety features that, unfortunately, sometimes contribute to catastrophic accidents. Perrow cites examples of so-called safety features as part of the problem in nuclear plants, marine accidents, and space travel [p. 260]. At the outset, Perrow suggests that “…no matter how effective conventional safety devices are, there is a form of accident that is inevitable.” [p.3]. The conclusion is, “...an expression of the integral characteristics of the system, not a statement of frequency” [p. 5].

Various investigators have advanced the idea that the mammalian dive response (MDR), also known as the dive or diving reflex, might be a cause, or mechanism of sudden unexpected death (SUD), particularly among young people. It is normally a safety feature of the body, but if and when several factors interact in unanticipated ways, then that safety feature may contribute to SUD.

## The Human Body as a Complex Tightly Coupled System

I suggest that some, but only some, cases of SUD are what Perrow might call a “normal accident” or “system accident.” Although, the risk is small at any moment during a human lifespan, unanticipated convergence of otherwise innocuous factors may lead to catastrophic failure of the system; sudden, unexpected, and premature death of an individual. In such cases, death is likely to result from processes that happen very fast, and can’t be turned off.

The resemblance of SUD to the Perrow view of system accidents in high-risk technological systems is striking. The risk of a catastrophic failure at any given moment is low, but inherent in the system. Convergence of factors or events not anticipated in the design (natural selection), construction (development), or operation (lifestyle) of the tightly coupled system (human body), may result in activation, or hyperactivation, of a prominent “safety feature” (the MDR) of the body and, rarely, SUD.

## The Mammalian Dive Response

The MDR is probably the most widely studied member of a family of oxygen conserving reflexes characterized by co-activation of parasympathetic and sympathetic divisions of the autonomic nervous system (ANS). These include chemoreceptor, diving, oculocardiac, somatic and nociceptor reflexes (Paton et al., 2005) as well as the trigeminocardiac reflex (Schaller et al., 2017). Named for different locations and/or mechanisms of activation, these reflexes combine parasympathetically mediated bradycardia with sympathetically mediated positive ventricular inotropy and automaticity (Braga et al., 2007). Thus, as noted by Paton et al. (2005), “From a clinical point of view it is important to recognize that in some instances, high drive to the heart from both autonomic limbs may be arrhythmogenic”. In the present communication, I use the term “the MDR” to refer to the potentially arrhythmogenic condition created by co-activation of parasympathetic and sympathetic divisions of the ANS as caused by one or more of the oxygen conserving reflexes The MDR is normally a safe, effective, and life-preserving safety feature. Most swimmers may engage in relatively long periods of breath-holding, exposure to cold water, and diving to considerable depths without incident. Among competitive or open water swimmers, the most likely cause of fatal drowning has been hypothesized to be arrhythmias (Asplund and Creswell, 2016). Mechanisms of fatal drowning among less experienced individuals in water have not been quantified, although some potentially arrhythmogenic risk factors have been identified (Vincenzi, 2018).

The potentially arrhythmogenic instances mentioned by Paton et al. (2005) may promote SUD in several different situations. These include some cases of sudden infant death syndrome (SIDS) (Lobban, 1991; Matturri et al., 2005) sudden unexpected death in epilepsy (SUDEP) (Vega, 2018), and fatal drowning caused by sudden cardiac death (SCD) in water (Vincenzi, 2018). I refer to SCD in water by the acronym SCDIW. In SCDIW, the diving reflex is the obvious initial candidate, but with apnea and subsequent hypoxia, the chemoreflex will also be activated. In SIDS and SUDEP, apnea and acute hypoxia probably trigger the chemoreflex, and possibly an “augmented” MDR (Vega, 2018). It is hypothesized that the MDR is involved in some cases of SIDS, SUDEP, and SCDIW, but acknowledged that additional or other reflexes might be involved in particular cases of SUD.

The MDR is variably expressed in humans and is generally more active in youth (Kaijser and Sachs, 1985; Goksör et al., 2002). Immersion or submersion in water results in bradycardia (Hurwitz and Furedy, 1986; Foster and Sheel, 2005; Lazar et al., 2013; Shamsuzzaman et al., 2014) and peripheral vasoconstriction. The MDR thus conserves oxygen by shunting blood to heart, lungs, and brain (Craig, 1968; Schaefer et al., 1968; Schagatay, 2011). In spite of profound bradycardia and decreased cardiac output, blood pressure is increased (Ferrigno et al., 1997; Willie et al., 2015), and cerebral blood flow is maintained or increased (Kjeld et al., 2009; Willie et al., 2015). In addition, the spleen contracts and provides additional red blood cells (Hurford et al., 1990; Bakovic et al., 2005; Schagatay et al., 2007). Conservation of oxygen allows survival under water for more than 10 min in trained, breath-hold divers (Hurford et al., 1990). The shift of blood to the central organs may result in pulmonary edema and hemoptysis, particularly among competitive breath-hold divers (Adir et al., 2004; Fitz-Clarke, 2006; Lindholm et al., 2008; Grünig et al., 2017). Sympathetic tone to the heart is increased, but because parasympathetic inhibition of the normal pacemaker of the heart predominates (Olsen et al., 1962; Wolf, 1964; Stromme et al., 1970; Lindholm and Lundgren, 2009; Alboni et al., 2011), the MDR has been called “diving bradycardia” (Stromme et al., 1970; Alboni et al., 2011).

Foster & Sheel reviewed the human diving response (Foster and Sheel, 2005). Inputs for the MDR include facial cold receptors, carotid chemoreceptors, baroreceptors, pulmonary stretch receptors, and atrial receptors. Foster and Sheel view apnea as a “master switch” that brings forth the coordinated effects that constitute the MDR. Their review cites both early and recent experimental observations by many investigators to create a comprehensive model for understanding the physiology of the MDR (Foster and Sheel, 2005).

Facial cold or facial immersion, even in the absence of bodily immersion or submersion, may elicit the MDR (Campbell et al., 1969; Gooden, 1972; Hurwitz and Furedy, 1986; Foster and Sheel, 2005). In a small subset of neonates, a short burst of warmth in the facial region also elicits the MDR (Smith et al., 1976; Allen et al., 1979). Activation of the MDR by facial immersion facilitated its experimental observation in many laboratories (Elsner et al., 1971; Hurwitz and Furedy, 1986; Wittmers et al., 1987). Even without submersion, apnea triggers the MDR. Apnea normally occurs when the body is submerged in water and hypoxia, even in the absence of facial cold or bodily immersion/submersion, rapidly activates the chemoreflex (Braga et al., 2007). It is well documented that a robust response is typically elicited by combining apnea and facial immersion (Elsner et al., 1966, 1971; Campbell et al., 1969; Hurwitz and Furedy, 1986; Shamsuzzaman et al., 2014).

Potentially pathological arrhythmias are common in humans during activation of the MDR (Hughes et al., 1981). Part of the increased automaticity of the ventricular myocardium during the MDR is due to catecholamines released into the circulation (Foster and Sheel, 2005). If SUD occurs as a consequence of activation of the MDR, then it is usually due to a fatal cardiac rhythm. Because SCD does not leave characteristic pathology at autopsy, many such cases remain unexplained.

Shattock and Tipton used the term autonomic conflict to refer to interaction of the “cold shock response” with the bradycardia of the MDR (Shattock and Tipton, 2012). The cold shock response involves activation of sympathetically driven tachycardia and thus promotes fatal arrhythmias responsible for deaths that may have been ascribed to drowning or hypothermia (Shattock and Tipton, 2012). Simultaneous activation of parasympathetic and sympathetic innervation of the isolated sino-atrial node (which could be viewed as *in vitro* autonomic conflict) results in an immediate and profound inhibition of automaticity followed by acceleration when the stimulus is terminated (Vincenzi and West, 1963). Thus, the parasympathetic response dominates but is terminated more rapidly than the sympathetic responses. A similar pattern was reported by Paton et al. (2005).

Shamsuzzaman et al. (2014) examined activation of the ANS during simulated diving experiences (simultaneous facial cold and apnea). Importantly, they demonstrated that autonomic responses were greater during simultaneous facial cold and apnea than with facial cold or apnea alone. During the late phase of simulated diving (greater than 30 s) they documented activation of sympathetic nerve outflow and decreased heart rate (Shamsuzzaman et al., 2014) setting up conditions for ventricular arrhythmias. The risk of a fatal rhythm is increased if the QT interval of the electrocardiogram is prolonged, either by inherited long QT syndrome (LQTS) (Ackerman et al., 1999; Choi et al., 2004; Tester et al., 2005a,^2011^) or drug-induced long QT syndrome (Vincenzi, 2016).

Many original articles and reviews reinforce the importance of the MDR as an important safety feature of the mammalian body (Scholander, 1963; Elsner et al., 1966, 1971; Gooden, 1972; Lindholm et al., 1999; Lindholm and Lundgren, 2009; Panneton, 2013). In spite of its importance, it was noted by Vega that the MDR is “Mostly unknown to physicians…” (Vega, 2018). That observation matches my own experience. Therefore, one of the goals of the current communication is to increase awareness of this vital reflex.

## The Hypothesis

The idea that MDR might contribute to death is not new (Wolf, 1964; Shattock and Tipton, 2012). The hypothesis to be advanced now is that in some cases of SIDS, SUDEP, and SCDIW, death results from activation of the MDR triggered by some combination of events or factors. In this view, the MDR is not the “cause” of death, but rather a final common mechanistic pathway. The multiple rare and/or unanticipated and unexpected factors or events (sometimes called causes) that interact to trigger the MDR differ in the various potentially fatal scenarios. Therefore, as in high-risk technological systems (Perrow, 1999), the search for a single “cause” of catastrophic failure is unlikely to be definitive and, a built-in safety feature is part of the problem.

The so-called triple risk model for understanding SIDS reflects an issue common to all three of the sudden death scenarios to be considered. That is, no single “cause” of SIDS (or SUDEP or SCDIW) has yet been identified. This is still the case in spite of many widely divergent and thoughtful suggestions. The common thread is that catastrophic failure results when unexpected factors interact. A likely sequence is that activation (or hyperactivation) of the MDR (rarely) results in SCD (Wolf, 1964; Lobban, 1995). According to the current hypothesis, no single cause will be found in these examples of SUD. The catastrophic failure known as SIDS is the result of multifactorial interactions (Guntheroth and Spiers, 2002). Thus, although the “back to sleep” program reduced SIDS deaths, it did not eliminate them. In other words, prone sleeping is one risk factor, but not “the cause” of SIDS (Goldberg et al., 2018).

Vega recently advanced an interesting hypothesis that appears to account for most cases of SUDEP following generalized tonic-clonic seizures (GTCS) (Vega, 2018). “Augmented” activation of the MDR followed by SCD is the proposed mechanism. It is a case of the MDR being activated even in the absence of water. The MDR may be a mechanism of SUDEP with or without activation by water, and the generally accepted definition of SUDEP excludes drowning (Hesdorffer et al., 2011) although the incidence of fatal drowning among epileptic patients is much greater than the general population (Holst et al., 2013).

There is no single cause of fatal drowning. As with SIDS, prevention programs that identify some of the risk factors help to reduce but do not eliminate fatal outcomes in water (Peden et al., 2016, 2017; Franklin et al., 2017). In many cases, interaction of genetic (Tester et al., 2005a), alcohol (Plueckhahn, 1984; Lunetta et al., 2004; Ahlm et al., 2013; Pajunen et al., 2017; Vincenzi, 2018) or drug (Pajunen et al., 2017) factors results in a fatal arrhythmia, strongly implicating the MDR.

There have been substantial contributions over a number of years by many different authors and groups trying to identify the cause(s) of sudden death in SIDS, SUDEP, and SCDIW. Such contributions support the hypothesis that interactions of a wide range of often unknown or unanticipated factors set the stage for catastrophic failure of a human life.

## Sudden Infant Death Syndrome (SIDS)

SIDS is defined as the SUD of an infant less than 1 year of age, with the onset of the fatal episode apparently occurring during sleep, that remains unexplained after a thorough investigation, including performance of a complete autopsy and review of the circumstances of death and the clinical history (Krous et al., 2004). SIDS is a major cause of death among children before the age of 1-year (Baruteau et al., 2017). The cause(s) remain unknown, particularly since SIDS strikes babies that appear to be healthy. The lack of specific pathological findings in SIDS cases created skepticism and allegations of infanticide (Bergman, 1997). Bergman and his collaborators, including JB Beckwith, emphasized that SIDS is not a will-o’-the-wisp killer, but a “definable disease” in need of testable hypotheses. They cited postmortem pulmonary edema, petechial hemorrhages in the thorax, and mild airway inflammation as evidence (Bergman et al., 1971). Subsequently, Beckwith and others noted that “Minor respiratory system infiltrates are acceptable, intrathoracic petechial hemorrhage is a supportive but not obligatory or diagnostic finding” (Krous et al., 2004). It is now understood that the shift of blood to the central compartment during activation of the MDR accounts for such findings in many cases, not only in SIDS, but also in SUDEP (where it is a characteristic finding) and in fatal drowning or competitive breath-hold diving (Linér and Andersson, 2008), where it is known as swimming-induced pulmonary edema (SIPE) (Grünig et al., 2017).

Many points of view have been put forward regarding “the cause” of SIDS, but no cause has been established. Some have come to adopt a so-called triple risk model for understanding SIDS (Goldberg et al., 2018). The model includes convergence of risk factors that include (1) a critical development period, (2) some exogenous stressor or stressors, and (3) a vulnerable infant. Early on, the triple risk model (Filiano and Kinney, 1994) was considered too restrictive by Guntheroth and Spiers who suggested multifactorial causation and interaction of risk factors with variable probabilities (Goldberg et al., 2018). That is a point of view favored by the current author. In short, the interaction of multiple unanticipated, or unknown factors leads to a sudden catastrophic failure. As will become apparent, those multiple factors may differ from case to case. Spinelli et al. (2017) reviewed the triple risk model and noted that the triple risk model was not designed to focus on a single cause of SIDS but rather to emphasize the complexity of the syndrome. Thus, “...SIDS deaths are not due to a single common pathway but instead involve the integration of known (and likely unknown) risk factors, environmental trigger events and underlying vulnerabilities” (Spinelli et al., 2017). According to the hypothesis being advanced here, activation of the MDR by such factors leads to SCD as the mechanism of death in some, but not all cases. Possible involvement of the MDR in SIDS has previously been advanced by others (Smith et al., 1976; Allen et al., 1979; Lobban, 1991, 1995).

Various investigators have emphasized some particular factor as the cause of SIDS. Each proposal has some merit, but none explains all cases. Few accounts clarify the mechanism of death. A more or less chronological consideration of publications reflects a shift of focus from organ level pathophysiological factors toward, neurological, cellular, immunological, and genetic factors as the cause of SIDS (or SUDEP or SCDIW).

A short burst of warm (25 C, 77 F) air over the ophthalmic or maxillary division of the trigeminal nerve resulted in apnea and a slight increase in heart rate in nearly all infants. However, a small percentage of infants responded with apnea and bradycardia (Smith et al., 1976; Allen et al., 1979). Such outlier responders are probably at greater risk of SIDS from overheating than their cohorts, possibly, but not necessarily, because of genetic differences. Warm air activation of the MDR may be rare (as is SIDS) because facial cold, is the usual trigger of MDR, at least in adults (Schuitema and Holm, 1988). Deaths from SIDS are more common in winter (Guntheroth et al., 1992), when even a very cold wind can produce a temporary apnea and bradycardia (Wierzba et al., 2011). Thus, facial cold as a trigger of MDR might also lead to sudden death in some cases (Schuitema and Holm, 1988). In other cases, overheating of the infant is the apparent trigger (Guntheroth and Spiers, 2001). Prone sleeping is a risk factor for SIDS because the face is a major site of heat loss for a swaddled or well-insulated infant. The incidence of SIDS was reported to increase on hot days in Montreal, Canada (Auger et al., 2015), but not in Vienna, Austria (Waldhoer and Heinzl, 2017). In any event, as early as the late 1970s it was concluded that further research was needed to explore the possible relationship between SIDS and a hyperactive diving reflex in the neonatal period (Smith et al., 1976; Allen et al., 1979; Lobban, 1991).

Goksör et al. (2002) examined the MDR in 36 infants, 2–12 months of age, during ordinary swimming. All subjects exhibited immediate and robust bradycardia with “dives” lasting between 2 and 8 s. The average heart rate decrease among infants 4–5 months of age, the most vulnerable period for SIDS, was 38.8%. Responses became less robust in successive months and the average decrease in heart rate among infants 10–12 months was 18.8% (Goksör et al., 2002). The MDR may be protective during a relatively hypoxic fetal life (Lavezzi, 2015), and protect the brain from hypoxia during the birthing process (Wierzba et al., 2011).

Singh et al. (2016) considered the MDR to be a peripheral subtype of the trigeminocardiac reflex, and one possible mechanism of SIDS. It was suggested that serotonergic and/or cholinergic dysfunction in the brain stem causes an exaggerated trigeminocardiac reflex with profound bradycardia, apnea, and death (Singh et al., 2016). Variations in neurotransmission might be a “modified blueprint” (mutation), or altered construction (epigenetic influences). Unanticipated interactions of such variations with other risk factors or events probably trigger SIDS in some cases.

A potential role of infections and inflammation in SIDS is apparent (Guntheroth et al., 1992; Goldwater, 2015; Goldwater, 2017). Goldwater focused on “…infection and sepsis as the only plausible mechanism of causation of sudden infant death syndrome” (Goldwater, 2017). Fever can result in overheating and rapid respiration, particularly in well-insulated infants. Hyperventilation rapidly causes hypokalemia (Edwards et al., 1977) and prolonged QT (Kannivelu et al., 2013) which, when coupled with the MDR, increases the risk of SCD (Vincenzi, 2018). Thus, some SIDS deaths may be similar to the hypoxic blackout phenomenon (see below) (Pearn et al., 2015).

Failure to arouse from sleep may be responsible for some cases if SIDS (Horne, 2018). A connection between the amount, duration and type of sleep apnea and near miss or actual SIDS events has been suggested (Schulte et al., 1982), possibly related to neuropathophysiological control of breathing during sleep (Sawaguchi et al., 2004). Neuropathology may be an underlying factor in some cases (Filiano and Kinney, 1994; Lavezzi, 2015), but the links to death are not clear. Absorption of nicotine or some byproducts may cause abnormal development of nuclei that control respiration (Lavezzi, 2015). Brainstem abnormalities of various transmitters and receptors have been reported in SIDS (Muhammad et al., 2018), including substance P and its neurokinin-I receptor, microdysgenesis of the hippocampus, and decreased orexin in various areas of the brain (Bright et al., 2018). Potential markers of the “precise cause” of SIDS are typically found in only a minority of SIDS cases, and may also be found in infants who died in other ways.

Prolonged QT, which increases the risk of SCD when combined with bradycardia, has been explored and is associated with some, but not all SIDS cases (Schaffer et al., 1991; Schwartz et al., 1998; Van Norstrand and Ackerman, 2010; Sarquella-Brugada et al., 2016; Baruteau et al., 2017; Tester et al., 2018). Like most issues involving SIDS, this has been controversial. In ECGs of 8 infants who subsequently died of SIDS, QTc and RR intervals were greater than controls, but the authors concluded that prolonged QT interval is not a risk factor (Weinstein and Steinschneider, 1985). ECGs were recorded from more than 7,000 newly born infants from two maternity hospitals in England. Fifteen of these infants subsequently died of SIDS. None showed prolonged QT that would meet the criteria of long QT (Southall et al., 1986). By contrast, based on measurements of over 44,000 infants in Italy, there was a significant difference in the QT interval of infants who subsequently died of SIDS, compared with those who died of other causes (Schwartz et al., 2009). Perhaps some gene pool difference accounts for the results. In any event, it appears likely that a small percentage of SIDS cases is related to LQTS. Whether infants should be screened and/or treated on the basis of QT interval is controversial (Guntheroth and Spiers, 1999; Hodgman and Siassi, 1999; Hoffman and Lister, 1999).

An ultra-rare genetic variant that may have contributed to arrhythmic sudden infant death was found in at most 14% of SIDS cases (Baruteau et al., 2017). Sarquella-Brugada et al. posed the question of whether SIDS is only a matter of genes encoding ion channels. They estimated that 10–15% of SIDS cases are caused by channelopathies, mainly LQTS and catecholaminergic polymorphic ventricular tachycardia (CPVT) (Sarquella-Brugada et al., 2016). Gray et al. (2018) concluded that there was no evidence for a monogenic basis for SIDS amongst non-cardiac genes. Experiments in mice exposed to neonatal hypoxia altered cardiac gene expression without mutation (Neary and Breckenridge, 2013). Less than 15% of more than 400 SIDS cases had a potentially informative variant in a heart disease-susceptibility gene when subjected to whole exome sequencing (Tester et al., 2018). There was over-representation of ultra-rare, nonsynonymous variants of cardiac channelopathy genes among SIDS infants of European ethnicity (Tester et al., 2018). At least 5 different pathways (and genes independently verified as genetic risks for SIDS) have been examined: serotonin transporter gene, cardiac channelopathies (3 different genes), immune dysfunction (4 different genes) metabolism (1 gene), nicotine response (no gene identified, but the risk of nicotine exposure is apparent). It was concluded that there is no justification for universal infant genetic testing to identify infants who may be at risk of SIDS. More work is needed to examine the mechanisms by which such variants contribute to the part of the triple risk that is labeled “infant vulnerability” (Van Norstrand and Ackerman, 2010).

A review of possible mechanisms of SIDS considered a wide variety of hypotheses including apnea (several subtypes), neuropathology, gastro-esophageal reflux, sleeping position, cardiac arrhythmias, infection, immunological factors, hyperthermia, inborn errors of metabolism, and endocrine abnormalities (Byard, 1991). It was proposed that SIDS is not related to cardiac issues and may be due to CNS or pulmonary causes (Gillette and Garson, 1992). Siren reviewed his SIDS-critical diaphragm failure hypothesis and emphasized that SIDS is multifactorial, but that critical diaphragm failure is the terminal event (Siren, 2016).

The diversity of proposed causes of SIDS is sobering. It is reminiscent of the parable of blind men describing the elephant. Each may have something to contribute, but none has the complete picture. While some of these ideas have faded from prominence, others, particularly genetic mutations, have emerged. However, SIDS is not “solved.” There is no single solution. In 1997 Guntheroth and Spiers urged more emphasis on the back-to-sleep campaign and criticized the suggestion by Bergman (1997), that more effort should be devoted to basic research (Guntheroth and Spiers, 1997). It would appear that both points of view were right. It is suggested that a multifactorial view of risks and potential mechanism(s) which includes the MDR will increase our insights might eventually reduce the number of tragic SIDS deaths.

## Sudden Unexpected Death in Epilepsy (SUDEP)

SUDEP is an autopsy- and toxicology-negative sudden death that occurs in a patient with epilepsy–a death, whether witnessed or not, that is unexpected. It should be noted that fatal drowning of an epileptic individual is not generally included in SUDEP (Holst et al., 2013). That may not be appropriate. If a patient with epilepsy happens to experience a GTCS while in or near water, then the death may be caused by a fatal arrhythmia and yet be classified as a (fatal) drowning. Drowning may appear to be the obvious cause of death in water, and a fatal arrhythmia leaves no telltale damage. Nashef and Ryvlin (2009) discussed various issues regarding the definition of SUDEP.

Epilepsy patients experience sudden death much more frequently than the general population (Hesdorffer et al., 2011). As with sudden unexpected fatal drowning, SUDEP occurs mainly in young individuals 12–14 years of age, when the MDR is quite active. The mechanism of SUDEP is not certain (Langan, 2000). Risk factors for SUDEP found to be statistically significant include GTCS, polytherapy with anti-seizure medications, the duration of epilepsy (longer, greater risk), a young age of onset, gender (male risk greater than female), symptomatic etiology (lower risk associated with idiopathic/cryptogenic and idiopathic generalized epilepsy), and lamotrigine therapy (increased risk) (Holst et al., 2013). Lamotrigine inhibits potassium currents mediated by the hERG channel (Danielsson et al., 2005) and may cause QT prolongation and thus increase the risk of fatal arrhythmias (Vincenzi, 2016). It remains to be seen if lamotrigine is a risk factor for SUDEP because of QT prolongation or perhaps some other mechanism (Nashef and Ryvlin, 2009).

Clinically relevant mutations in cardiac arrhythmia and epilepsy genes were reported in 46% of 61 SUDEP cases (Bagnall et al., 2016). An unsurprising common thread is that a number of SUDEP victims (and SCDIW and SIDS victims) carried a variety of mutations that cause long QT. Many also carried mutations that increase the risk of CPVT, a different route to a fatal cardiac rhythm. Thus, not all of the sudden deaths, even SCDs within a defined scenario, are related to a single mechanism. As with long QT, there are a variety of mutations that underlie CPVT (Bagnall et al., 2016). This is in addition to a host of non-genetic risk factors (Holst et al., 2013).

Apnea often occurs during various types of seizures (Nashef et al., 1996). Cardiovascular changes consistent with activation of the MDR may or may not be seen. Ictal bradycardia commonly produces cardiogenic syncope (Reeves et al., 1996). An increase in heart rate was seen in 91% of monitored seizures; most of which were not GTCS (Ryvlin et al., 2013a). SUDEP is associated mainly with GTCS and only rarely with other types of seizures (Nashef et al., 1998; Langan, 2000; Holst et al., 2013; Ryvlin et al., 2013a). In a few cases in which SUDEP occurred during monitoring, there was postictal hypopnea followed by apnea, bradycardia, and asystole with a 40% recurrence rate of ictal asystole (Hampel et al., 2017). Nashef and Ryvlin (2009) noted that “Different risk factors and mechanisms may operate with a final common pathway of cardiorespiratory failure”.

Vega hypothesized that individuals with GTCS are particularly prone to sudden death as a consequence of an “augmented” MDR (Vega, 2018). This augmented “dive reflex,” activated in the absence of water immersion is presumably triggered by hypoxia secondary to apnea coupled with a rapid increase in the demand by muscle for oxygen. It is an example of unexpected factors interacting and triggering a physiological safety feature, the MDR. While skeletal muscle can revert to anaerobic metabolism, circulation to the brain must be preserved. Blood is shifted to the central compartment, and cerebral blood flow is usually maintained (Palada et al., 2007; Joulia et al., 2009; Cross et al., 2014; Willie et al., 2015). Unfortunately, that may not always be the case in SUDEP. Vega noted, “Theoretically, a MDR preceded by intense hyperventilation (i.e., prolonged apnea preceded by hyperventilation), such as that seen in the majority of SUDEP cases reported by Ryvlin et al. (2013b), could further exacerbate this process by lowering arterial CO_2_ levels, and thus inducing cerebral vasoconstriction before ictal MDR deployment” (Vega, 2018). Another mechanism may also contribute to SUDEP as a consequence of hyperventilation followed by activation of the MDR. Hyperventilation causes QT prolongation within 1 min (Kannivelu et al., 2013), and the bradycardia of the MDR increases the risk of both Torsade de Pointes (TdP) and ventricular fibrillation (VF) when QT is prolonged (Vincenzi, 2018).

Autonomic dysfunction during or after seizures may contribute to SUDEP (Devinsky, 2004; Drake et al., 1998). Somewhat like the WHO definition of drowning that focuses on impaired respiration (van Beeck et al., 2005), Devinsky concluded that pulmonary dysfunction, including pulmonary edema, was important and that cardiovascular effects are not the cause of SUDEP (Devinsky, 2004). However, Vega pointed out that drastic cardiovascular changes needed to maintain oxygen delivery to the brain can result in many of the pathological hallmarks of SUDEP including pulmonary edema and hemorrhage. Pulmonary edema and hemorrhage associated with SUDEP (and SIPE and SIDS) depends a profound shift of blood volume to heart, lungs, and brain mediated by the MDR (Craig, 1968; Schaefer et al., 1968; Schagatay, 2011). The MDR protects the brain from hypoxia while the peripheral organs switch to anaerobic metabolism. Rarely, sudden death may happen. More usually, during recovery, there is an increase in lactic acid in the blood – as blood is returned to the systemic circulation from transiently anaerobic organs (Vega, 2018). For the same reasons, a decrease in blood pH is also seen following competitive breath-hold diving (Ferrigno et al., 1997).

If apnea is coupled with exercise, then the MDR response is even more vigorous (Stromme et al., 1970; Andersson et al., 2002; Palada et al., 2007). Therefore, Vega proposed that during GTCS (when apnea and vigorous muscular activity coincide with a loss of consciousness), MDR is “augmented” and is not subject to attenuation by mental processes (Ross and Steptoe, 1980). SUDEP is catastrophic failure of a tightly coupled human body system confronted with the rare challenge of simultaneous apnea, extreme muscular activity, hypoxia, and loss of consciousness. The MDR is triggered, and the result is a fatal arrhythmia. It is a system accident ala Perrow (1999).

Epilepsy is associated with fatal drowning at an estimated rate of nearly ten times that of the general population (Bain et al., 2018). Most such fatal drownings occur in the bathtub. Like other SUDEP cases, most occur in young persons in whom the MDR is quite active (Kaijser and Sachs, 1985). By contrast, in the general population, 60% of bathtub drownings occur in persons 65 years of age or older (Bain et al., 2018). A natural assumption is that an inability to coordinate lifesaving movement forms the basis for the increased risk of fatal drowning during a seizure. However, some of the reported “drownings” occurred in a bucket, sink or small water-containing vessel. It seems reasonable to suggest that “augmented” MDR associated with GTCSs (Vega, 2018) may be further augmented when a seizure occurs during water-related activities; with or without facial immersion. It is suggested that a “doubly-augmented MDR” substantially increases the risk of a death classified as a fatal drowning rather than SUDEP.

## Sudden Cardiac Death in Water (SCDIW)

The World Health Organization (WHO) defines drowning as, “respiratory impairment due to submersion or immersion in liquid,” without reference to the outcome (death, morbidity, or no morbidity) (van Beeck et al., 2005). Commonly, the word drowning means death in water. Fatal drowning is now the preferred terminology. Arrhythmias have been hypothesized to be the most common cause of fatal drownings of competitive swimmers (Asplund and Creswell, 2016). Individuals with few or no swimming skills presumably die commonly by respiratory impairment. Thus, not all sudden unexpected fatal drownings are cases of SCDIW. Both mechanisms of death are most often classified merely as drowning. In the present communication, I focus mainly on the role of the MDR in SCDIW.

The MDR as a diving reflex is, indeed arrhythmogenic. Face immersion with breath-hold for as long as possible was performed by healthy late adolescents while monitoring the ECG. One of 20 subjects did not complete the protocol because of heterogenous ventricular extrasystoles. Actually, 20% of the subjects exhibited ventricular extrasystoles, especially late in the protocol. Arrhythmias were difficult to predict due to high interindividual variability (Wierzba et al., 2011).

SCDIW is triggered by the interaction of certain risk factors with the MDR. One such risk factor is a prolonged QT interval. QT prolongation may result from congenital, drug, or metabolic causes. Among children with nonfamilial long QT, facial immersion in cold water further increased QT and was suggested as a possibly useful predictor of elevated risk of fatal drowning (Yoshinaga et al., 1999). Prolonged QT increases the risk of TdP and SCD due to VF (Roden, 2004; Straus et al., 2005, 2006; De Bruin et al., 2007; Koene et al., 2017). However, the risk of SCD due to QT prolongation alone is relatively small, and many individuals live normal lives without being aware of inherited LQTS. The risk of death is greatly increased when LQTS is combined with swimming (Keating et al., 1991; Ackerman et al., 1999; Yang et al., 2002; Tester et al., 2005a; 2005b, 2011). When QT prolongation interacts with bradycardia of the MDR elicited by a water-related activity, then a fatal cardiac rhythm is likely to result in SCDIW (Vincenzi, 2018). The prevalence of congenital prolongation of QT interval is estimated to be about 1:2000 and would not be subject to much elimination by natural selection (Schwartz et al., 2009).

Because defibrillation may restore circulation in an individual who just experienced SCD, including SCDIW, it is essential that recreational and competitive swimming venues have rapid access to automatic external defibrillators and personnel trained in their use. The same is true for other athletic venues where SCD may occur in response to a variety of triggers (Sweeting and Semsarian, 2018).

Familial association with fatal drowning was noted before the advent of affordable genetic analysis (Harris et al., 1992). Now it is clear that some cases of SCDIW are associated with a change in the “system blueprint,” i.e., congenital LQTS (Ackerman et al., 1999). Many different mutations underlie LQTS (Tester et al., 2005b; Smith et al., 2016; Wu et al., 2016). Likewise, QT prolongation may be due “operation of the system” (e.g., exposure to alcohol and/or a prescribed drug). Over 200 medically prescribed drugs cause QT prolongation and, to a greater or lesser extent, increase the risk of a fatal rhythm when combined with bradycardia (Roden, 2004; Woosley et al., 2017).

Metabolic changes, particularly electrolyte imbalances, promote prolongation of the QT interval (Harris et al., 1992). Hypokalemia is the strongest non-cardiologic factor associated with QT prolongation (Marill and Miller, 2017). Within one minute, hyperventilation causes QT prolongation (Kannivelu et al., 2013), related to changes in blood pH and hypokalemia (Edwards et al., 1977). Hyperventilation prior to a breath-hold dive may result in fatal drowning. The phenomenon is known as shallow water blackout, or hypoxic blackout (Craig, 1961; Pearn et al., 2015). The usual explanation is that hypocarbia produced by hyperventilation delays the urge to breathe beyond the time that oxygen is so depleted that consciousness is suddenly lost (blackout) (Pearn et al., 2015). An equally plausible interpretation is that acutely prolonged QT caused by hyperventilation, when coupled with diving bradycardia, causes SCDIW. In such a case, failure of delivery of blood to the brain rather than hypoxemia is the pathway to blackout. The two mechanisms of death would be indistinguishable upon autopsy.

About 60–80% of accidents in high-risk technological systems are due to operator error [p. 9]. One may suggest, somewhat tongue in cheek, that sudden accidental death of human beings, also involves a relatively high percentage of “operator error.” When combined with swimming, consumption of alcohol represents such an error. Alcohol is the drug most commonly associated with fatal drowning (Plueckhahn, 1984; Lunetta et al., 1998, 2004; Smith et al., 2001; Driscoll et al., 2004; Ahlm et al., 2013; Pajunen et al., 2017). Alcohol increases the sympathetic component of the MDR (Wittmers et al., 1987) and thus increases the likelihood of ventricular extrasystoles when the normal pacemaker is inhibited. Alcohol causes psychomotor impairment (Grant et al., 2000), and the usual assumption is that the victim of fatal drowning was simply “drunk” and unable to swim. But alcohol also increases QT and QT dispersion (Rossinen et al., 1999; Uyarel et al., 2005) and the risk of SCD. In a recent study of alcohol and drugs in unintentional fatal drowning victims, alcohol was present at levels above 0.05 mg/dL in about 60% of the cases (Pajunen et al., 2017). A number of QT-prolonging drugs, with or without alcohol, were also present and contributed to fatal drowning in about 14% of the cases (Pajunen et al., 2017). Until recently in our evolutionary history, the MDR was not paired with alcohol or prescription drugs. The simultaneous presence of such factors creates a mode of operation that was not “considered” in the design and construction of the mammalian body.

SIPE is the sudden development of pulmonary edema during strenuous swimming, particularly in cold water (Grünig et al., 2017). It is seen in competitive breath-hold competitive diving, particularly following deep dives (Lindholm et al., 2008). It is due to the shift in blood to the central compartment and hypertension mediated by the MDR (Adir et al., 2004). That shift, simultaneous with chest compression, exerts effects similar to restrictive heart disease (Marabotti et al., 2008). Most cases of SIPE include hemoptysis and dyspnea (Grünig et al., 2017). SIPE has been associated with fatal drowning, and it seems likely that some cases result from blackout secondary to hypoxemia, and some from SCDIW. The latter seems particularly likely when the MDR interacts with the cold shock response (Shattock and Tipton, 2012).

Sympathetic discharge triggered by alerting stimuli increases ventricular arrhythmias experimentally (Nalivaiko et al., 2004). Likewise, the cold shock response increases sympathetic discharge (Shattock and Tipton, 2012) and increases the risk of SCDIW upon interaction with diving bradycardia. This interaction may be important during the use of a self-contained underwater breathing apparatus (SCUBA). Diving to depth may bring the SCUBA diver into cold water, without hypoxia secondary to apnea but with the bradycardia of the classical dive reflex. Fatal drowning among SCUBA divers is probably increased by cold water, particularly in the absence of a wet suit or dry suit. The diving community knows that other risk factors such as pre-existing heart disease (Marabotti et al., 2013), alcohol, and drugs should be avoided. For example, a SCUBA diver at depth apparently experienced TdP that degenerated into VF. The postmortem level of a QT-prolonging drug was high, but that dose had been survived for over a year until she went diving (Vincenzi and Lunetta, 2015). Likewise, a SCUBA diver who had been taking high doses of amphetamine for some time apparently suffered a fatal rhythm at depth. When combined with the cold shock response, amphetamine and the MDR probably initiated a CPVT-like rhythm (Vincenzi, unpublished).

Few epileptic drownings are associated with alcohol or drugs. Some cases presumably occur as a consequence of activation of the MDR and a fatal rhythm not necessarily preceded by a seizure. Some fatal drownings of epileptic patients almost certainly occur as a consequence of augmented activation of the MDR triggered by a GTCS, as proposed by Vega (2018). Bagnall et al. (2016) reported that 7% of SUDEP cases had LQTS. That would make them at high risk for SCDIW (Tester et al., 2011; Vincenzi, 2018). It is suggested that such victims die as a result of typical immersion-activated MDR followed by SCDIW. A further 15% of SUDEP cases examined by Bagnall et al. (2016) had candidate pathogenic variants in genes associated with cardiac arrhythmias including genes associated with CPVT. CPVT may be a common cause of autopsy-negative SUD among athletes. Basketball players are at particularly high risk (Harmon et al., 2015). “Drowning” was the second most common cause of accidental death among athletes; a category that would have included SCDIW (Harmon, personal communication). Once again, one is faced with SUD for which there is no single cause.

Obviously, not all fatal drownings are the result of SCDIW. On the other hand, the preponderance of evidence would appear to support the following view. Rarely, but not always in the context of some water-related activity, a variety of factors and/or events, some unknown, some simply unexpected, may interact with each other to trigger the MDR with a resultant fatal cardiac rhythm. Probably among others, such factors, or events, include genetic, epigenetic, metabolic, environmental, and operational issues.

## Summary

Based on ideas put forth by Perrow (1999), the human body is considered here as a complex tightly coupled system in which the interaction of one or more of risk factors &/or events may result in catastrophic failure - SUD. The body as a system is “constructed” during fetal and postnatal development according to genetic “blueprints,” is “maintained” during life in the face of various challenges, and is “operated” in ways that may increase the risk of SUD in different potential death scenarios. Table 1 summarizes, reported blueprint, construction, maintenance, or operational features of the human body that may activate or interact with the MDR and contribute to SUD. Entries in each column are listed alphabetically to avoid implications of relative importance. Many different risk factors and events ranging from genetic mutations to life-style choices are potential contributors to SUD. Risk factors and events may happen at phases of life ranging from prenatal to potentially premortem. Rarely, interaction of the MDR with the risk factors and events leads to SUD. Unique features of the MDR in certain individuals may increase the risk of a fatal outcome. While there are many possible risk factors and events and some individual variations in the MDR responses, there are comparatively few mechanisms of SUD. Depending on circumstances, the result might be SIDS, SUDEP or SCDIW.

**Table 1:** SIDS, SUDEP, and SCDIW: multifactorial features of a complex tightly coupled system.

Blueprint	Construction	Maintenance	Operation
**Risk factors and events**			
CPVT mutations	Fetal nicotine	Acute long QT	Alcohol
Epilepsy^∗^	Neuropathology	Cold	Apnea
LQTS mutations	Prematurity	Epigenetics	Breath-hold
Mitochondrial variants	Vulnerable period	Hypokalemia	Cold shock response
Ultra rare mutations		Infection	Facial immersion
		Inflammation	Hyperventilation
		Overheating	Prone sleeping
			QT-prolonging drugs
			Swimming
			Tub bathing
**The MDR (co-activation of parasympathetic and sympathetic divisions of the ANS)**			
Natural selection	Brain stem	Acetylcholine	Blood shift
	Hippocampus	Serotonin	Bradycardia
		Substance P	Hypertension
			Pulmonary edema
			Spleen contraction
**Possible mechanism of sudden unexpected death**			
			Asystole
			CPVT
			Respiratory failure
			VF


*^∗^epilepsy may be genetic or acquired.*

SCD is not the only possible mechanism of death in SIDS, SUDEP, or SCDIW. For example, conventional wisdom, as well as the WHO definition of drowning, focuses on “respiratory impairment” (van Beeck et al., 2005). Likewise, ictal apnea is a common feature of epilepsy and in some cases may result in SUDEP due to respiratory failure. The triple risk model for understanding SIDS includes respiratory infections as extrinsic risk factors. In other words, impaired respiration might be the mechanism of sudden death in some cases of SIDS, SUDEP, or SCDIW.

The hypothesis put forth here is that in some cases of SIDS, SUDEP, and SCDIW, death results from activation of the MDR triggered by some combination of events or factors. The hypothesis will not be tested experimentally on humans but rather as experience on such deaths accumulates in light of increased awareness of the arrhythmogenic potential of the MDR. There is no single cause of such deaths although some researchers focus on finding one. A particular constellation of factors and/or events may be present or absent in a given case. Often such factors/events remain undetermined even following intense investigation including autopsy and forensic toxicology.

Complex technological systems contain inherent risks of catastrophic events in spite of efforts to prevent them. As Perrow put it, “…the normal accident generally (not always) means that the interactions are not only unexpected but are incomprehensible for some critical period of time” (Perrow, 1999). In the case of MDR-induced TdP/VF, that period of time may be very short. Perrow also noted that unanticipated interactions with a safety feature built into the system may lead to catastrophic failure. A 1963 article on the MDR in *Scientific American* was titled, “The Master Switch of Life” (Scholander, 1963). Perhaps it might have been titled, “The Master Switch of Life or Death.”

## Author Contributions

FFV conceived of and wrote the manuscript.

## Conflict of Interest Statement

The author declares that the research was conducted in the absence of any commercial or financial relationships that could be construed as a potential conflict of interest.

## Abbreviations

ANS: autonomic nervous system
CPVT: catecholaminergic polymorphic ventricular tachycardia
GTCS: generalized tonic-clonic seizure
LQTS: long QT syndrome
MDR: mammalian dive response
QT: QT interval of the electrocardiogram
QTc: QT interval of the electrocardiogram corrected for heart rate
SCD: sudden cardiac death
SCDIW: sudden cardiac death in water
SIDS: sudden infant death syndrome
SIPE: swimming induced pulmonary edema
SUD: sudden unexpected death
SUDEP: sudden unexpected death in epilepsy
TdP: Torsade de Pointes
VF: ventricular fibrillation
WHO: World Health Organization
